# Draft genome of *Thermomonospora* sp. CIT 1 (Thermomonosporaceae) and *in silico* evidence of its functional role in filter cake biomass deconstruction

**DOI:** 10.1590/1678-4685-GMB-2017-0376

**Published:** 2019-03-11

**Authors:** Wellington P. Omori, Daniel G. Pinheiro, Luciano T. Kishi, Camila C. Fernandes, Gabriela C. Fernandes, Elisângela S. Gomes-Pepe, Claudio D. Pavani, Eliana G. de M. Lemos, Jackson A. M. de Souza

**Affiliations:** 1 Universidade Estadual Paulista Universidade Estadual Paulista Programa de Pós-Graduação em Microbiologia Agropecuária JaboticabalSP Brazil Programa de Pós-Graduação em Microbiologia Agropecuária, Faculdade de Ciências Agrárias e Veterinárias, Universidade Estadual Paulista (UNESP), Jaboticabal, SP, Brazil; 2 Universidade Estadual Paulista Universidade Estadual Paulista Departamento de Tecnologia, Laboratório de Bioinformática JaboticabalSP Brazil Departamento de Tecnologia, Laboratório de Bioinformática, Faculdade de Ciências Agrárias e Veterinárias, Universidade Estadual Paulista (UNESP), Jaboticabal, SP, Brazil; 3 Universidade Estadual Paulista Universidade Estadual Paulista Laboratório Multiusuário Centralizado para Sequenciamento de DNA em Larga Escala e Análise de Expressão Gênica (LMSeq) JaboticabalSP Brazil Laboratório Multiusuário Centralizado para Sequenciamento de DNA em Larga Escala e Análise de Expressão Gênica (LMSeq), Faculdade de Ciências Agrárias e Veterinárias, Universidade Estadual Paulista (UNESP), Jaboticabal, SP, Brazil; 4 Universidade Estadual Paulista Universidade Estadual Paulista Departamento de Biologia Aplicada à Agropecuária Laboratório de Genética Aplicada JaboticabalSP Brazil Departamento de Biologia Aplicada à Agropecuária, Laboratório de Genética Aplicada, Faculdade de Ciências Agrárias e Veterinárias, Universidade Estadual Paulista (UNESP), Jaboticabal, SP, Brazil

**Keywords:** Actinobacteria, pectin, hemicellulose, crystalline cellulose, orthologous genes

## Abstract

The filter cake from sugar cane processing is rich in organic matter and nutrients, which favors the proliferation of microorganisms with potential to deconstruct plant biomass. From the metagenomic data of this material, we assembled a draft genome that was phylogenetically related to *Thermomonospora curvata* DSM 43183, which shows the functional and ecological importance of this bacterium in the filter cake. *Thermomonospora* is a gram-positive bacterium that produces cellulases in compost, and it can survive temperatures of 60 ºC. We identified a complete set of biomass depolymerizing enzymes in the draft genome of *Thermomonospora* sp. CIT 1, such as α-amylase, catalase-peroxidases, β-mannanase, and arabinanase, demonstrating the potential of this bacterium to deconstruct the components of starch, lignin, and hemicellulose. In addition, the draft genome of *Thermomonospora* sp. CIT 1 contains 18 genes that do not share identity with five other species of *Thermomonospora*, suggesting that this bacterium has different genetic characteristics than those present in genomes reported so far for this genus. These findings add a new dimension to the current understanding of the functional profile of this microorganism that inhabits agro-industrial waste, which may boost new gene discoveries and be of importance for application in the production of bioethanol.

A by-product of sugar manufacture, the filter cake is very rich in organic matter, phosphorus, moisture (70-80%), and other nutrients. The filter cake is a mixture of the decantation sludge with residual particles of sugarcane bagasse that results from the process of milling and extraction of the cane broth. Produced in large quantities, the filter cake can be used *in natura* as a substitute for commercial soil fertilizers (de Fravet *et al.*, 2010), or it can be stored in an appropriate place for up to 40 days before use in agriculture, making it an environmental conductive agent for the development of numerous microorganisms.

Wastes from agriculture and plant biomass composting are natural habitats of *Thermomonospora curvata* (Thermomonosporaceae) bacteria (Wang *et al.*, 2016) that can survive temperatures of 60 °C or above (Wei *et al.*, 2014). Actinobacteria have a slow growth rate and one of the most abundant microbial groups in composts, a fact related mainly to adaptive advantages related to lignocellulases production capacity and secretion of antimicrobial agents (Martins *et al.*, 2013; Wang *et al.*, 2016). However, these lignocellulases must be able to overcome the recalcitrance of plant biomass, allowing the release of sugars (pentoses and hexoses) and nutrients, which can be used in biological processes fundamental to cell maintenance. The recalcitrance of lignocellulose occurs due to cellulose paracrystallinity, molecular complexity of hemicellulose coating on cellulose microfibrils, and the interpenetration and encapsulation of polysaccharide components by lignin (Cragg *et al.*, 2015).

Enzymes capable of deconstructing lignocellulose are widely used in the pretreatment of plant biomass for the production of bioethanol, where the sugars derived from this process can be used in the fermentation. In fact, the use of enzymes in this type of process is the most indicated from the environmental and economic point of view, since, to overcome the recalcitrance of the plant biomass, it is necessary to perform severe chemical and/or physical pre-treatments (Klein-Marcuschamer *et al.*, 2012; Cragg *et al.*, 2015). This results in the production of undesirable by-products such as release of effluents of high toxicity to the environment that must be neutralized, which raises production costs. However, for the production of bioethanol to be implemented on a large scale and in a competitive manner, it is essential to reduce the cost of enzyme production and have an effective production schedule cocktail (in mg per ton) as well as the optimization of the enzymatic composition to increase efficiency through the discovery and kinetic characterization of new enzymes (Klein-Marcuschamer *et al.*, 2012).

*Thermomonospora curvata* (Thermomonosporaceae) has a genetic arsenal with great biotechnological potential (Wei *et al.*, 2014; Wang *et al.*, 2016). However, many studies did not adequately explore other characteristics of the genome of this microorganism, such as identification of genes related to lignocellulose degradation. In this context, materials rich in plant matter such as agro-industrial waste deposits favor the selection of genes with potential biomass deconstruction, creating a favorable environment for the discovery of new strains with new enzymes or unpublished genes. In this work, we report genes present in the draft genome CIT 1 that is phylogenetically related to *T. curvata* DSM 43183, which was reconstructed from sequences derived from total metagenome of filter cake.

In January 2013, two random samples were collected at 0-20 cm (393 mL) depth of the surface of the filter cake stack and stored for approximately 40 days. The location of the collection was Fazenda Itaquerê in Nova Europa, state of São Paulo, Brazil. Samples were transported immediately to the laboratory and after homogenization and formation of a composite, the sample was weighed to 250 mg of total metagenomic DNA extraction using Fast DNA^®^ kit for Soil (Bio 10, Quantum Biotechnologies), following the manufacturer’s instructions. The purity and concentration of the DNA were analyzed in Qubit^®^ 2.0 Fluorometer (Life Technologies) with the Qubit dsDNA BR Assay Kit (Invitrogen^®^), following the manufacturer’s recommendations. A sample of the filter cake was sent for bromatological analysis.

The metagenomic DNA sequencing library was prepared according to the TruSeq^®^ DNA Sample Preparation v2 (Illumina^®^) protocol, as per the manufacturer’s recommendations. Sequencing of the shotgun library (1 μg DNA) was performed on the Illumina^®^ platform, HiScanSQ equipment, using Paired-End Cluster Generation Kit v3 (Illumina^®^) and TruSeq SBS Kit v3 - 200 Cycles (Illumina^®^), following the recommendations of the manufacturer.

Removal of sequences with low quality (Q<20) and length less than 50 bp, as well as assembly of scaffolds, formation of genome clusters recovered from metagenomic data, closure of gaps to increase scaffold extension and verification of coverage sequencing were performed as previously described (Jourda *et al.*, 2015; Kishi *et al.*, 2015). At the end, 29 scaffolds were retrieved, which were annotated automatically on the online RAST server (Aziz *et al.*, 2008). The prediction of carbohydrase enzymes was done using the dbCAN server (database Carbohydrate-active enzyme ANnotation) (Yin *et al.*, 2012). In order to verify if the genes predicted by dbCAN were similar (best hits, ≥ 85% of coverage) to some carbohydrase gene deposited in GenBank, we performed a blastp search (Altschul *et al.*, 1990) against the non-redundant protein database of the National Center for Biotechnology Information (NCBI).

After searching the housekeeping genes of 19 bacterial species phylogenetically related to the draft genome CIT 1, the sequences were aligned separately using Mafft v.7.215 [Bibr B21] [Bibr B22] [Bibr B23] [Bibr B24] [Bibr B25] [Bibr B26] [Bibr B27] [Bibr B28](Katoh and Standley, 2013) and subsequently concatenated with Mesquite v.2.74 (Maddison and Maddison, 2017). The prediction of the best nucleotide substitution model, based on the lower Akaike Information Criterion correction (AICc) value, was performed with the online program IQ-TREE v.1.5.5 (Trifinopoulos *et al.*, 2016). For the construction of the phylogenetic tree, we used the IQ-TREE program by selecting the nucleotide substitution matrix with the lowest AIC, correction option for frequency states optimized for Maximum Likelihood (ML) and bootstrap with 1,000 replicates using the algorithm Ultrafast (Trifinopoulos *et al.*, 2016).

We used the online program OrthoVenn (Wang *et al.*, 2015) to estimate the orthologous genes and distinguish between the singularities of draft genomes of CIT 1 and *Thermomonospora curvata* DSM 43183 (Thermomonosporaceae, accession CP001738). To verify the proximity between genomes, we used the online software JspeciesWS v.3.0.11 (Richter *et al.*, 2016), which uses pairwise alignment between genome sequences to determine the percentage of similarity between organisms from the average nucleotide identity (ANI) parameters based on blast (ANIb), MUMmer (ANIm), and tetranucleotide signature correlation index (Tetra).

This Whole Genome Shotgun project has been deposited at DDBJ/ENA/GenBank under the accession MOYN00000000. The version described in this paper is MOYN02000000. In RAST server, the accession number is 471852.20.

After separating the genome groups of the metagenomic data of the filter cake, 101 scaffolds that were not similar (≤70% and coverage ≤80%) were identified and eliminated from the draft genome CIT 1 dataset of *Thermomonospora curvata* (Thermomonosporaceae) (Table S1). According to RAST platform, the draft genome CIT 1 presented a size of 5,460,082 bp, GC content of 71.8% and 4,834 coding sequences (CDS), data very similar to those observed in the circular curve genome of *T. curvata* DSM 43183 (Table S2). The total sequencing showed 49,554,712 paired-end reads, from where the draft genome was assembled from 9.48% of the reads, with 180X coverage. According to data from this same platform, the enzymes involved in carbohydrate metabolism were distributed among the central metabolism subsystems of carbohydrates (146), fermentation (73), polysaccharides (28), and monosaccharides (24), demonstrating the potential of using sugars derived from the plant fiber present in the filter cake.

The analysis in dbCAN for draft genome CIT 1 identified 170 candidate genes of the carbohydrases synthesis, whereas the same analysis performed on the *Thermomonospora curvata* DSM 43183 (Thermomonosporaceae) showed only 93 genes of the same group (Tables S3 and S4). Among these, genes of major interest for bioethanol production and little explored in the genome of *Thermomonospora* species are those related to the classes of glycoside hydrolases (GH) β-mannanase (GH26), arabinanase (GH43) e α-amylase (GH13) (Table S3). These enzymes act on various lignocellulosic structures (Figure 1A-D), and such structures were identified in the bromatological analysis of the filter cake in the proportions of 25% cellulose, 9% lignin, 8% hemicellulose, and 8% non-nitrogenated extract (pectin, starch, etc.).

**Figure 1:**
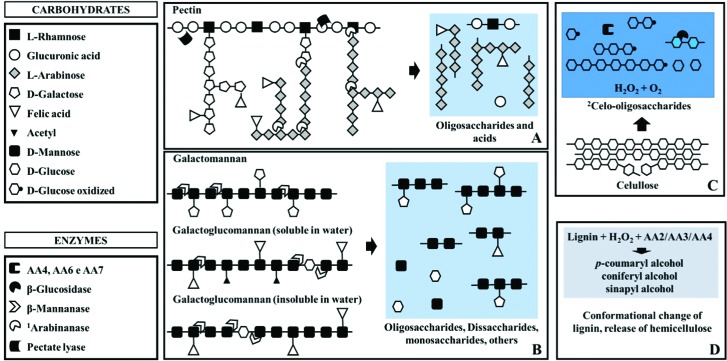
Identification of genes that can act on the main plant biomass structures in the filter cake present in the draft genome CIT 1. (A) Enzymes that act on pectin, releasing mainly glucuronic acid and polysaccharides. (B) Enzymes that act on the main constituents of hemicellulose, releasing oligosaccharides, disaccharides, monosaccharides, etc.. (C) Enzymes that can act on the cellulose, releasing components of cello-oligosaccharides and glucose. (D) mechanism of action of some Auxiliary Activities (AA) that act on the conformational change of lignin and availability of structural components with aromatic rings of this heteropolymer. ^1^According to the database BRENDA (https://www.brenda-enzymes.org/), acts best on linear 1,5-α-L-arabinan. Also acts on branched arabinan, but more slowly. ^2^In addition to cellobiose, AAs can oxidize other cello-oligosaccharides, such as hepta-cellobiose, trio-cellobiose, etc.

The 29 scaffolds of the draft genome CIT 1 were very similar (mean of 99%) to the circular genome of *Thermomonospora curvata* DSM 43183 (Thermomonosporaceae). Phylogenetic analysis allowed confirming the taxonomic positioning of the draft genome CIT 1 (100% bootstrap) as belonging to the species of *T. curvata* (Figure 2A). This classification is consistent with the high similarity (> 99%) between pairs indicated by the %ANIb, %ANIm and, Tetra indexes (Richter *et al.*, 2016) obtained with JSpeciesWS (Table 1). However, the analysis performed with OrthoVenn, showed that of the 3,756 clusters of orthologous genes present in the draft genome of *Thermomonospora* sp. CIT 1, only 18 clusters are not shared with any other species (Figure 2B and C). According to Gene Ontology (GO), these genes are mainly related to the function with hydrolases activity, acting on C-O, C-N and, C-C bonds (GO:0016787), peptidase activity (GO:0008233) and, genes related to the transport of macromolecules, small molecules, and ions through the cell wall (GO:0005215) (data not shown). Clusters related to transport of sugars through the membrane were also corroborated by Uniprot data (also via Ortho Venn) showing their importance to bioethanol production by biotechnology exploitation.

**Figure 2:**
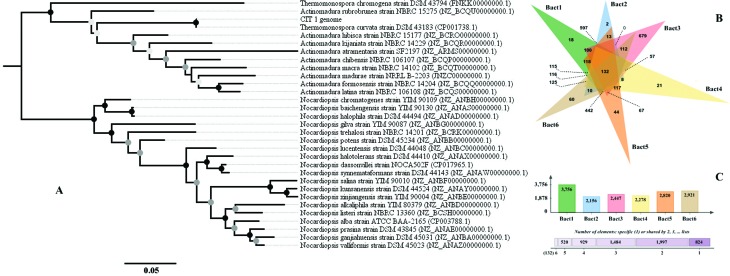
Phylogenetic analysis using Maximum Likelihood to verify the phylogenetic position of the draft genome CIT 1 (*Thermomonospora* sp. CIT 1) and identification of clusters of orthologous genes using Ortho Venn. (A) Phylogenetic tree demonstrating that the draft genome CIT 1 is related to the clade of *Thermomonospora curvata* DSM 43183 (Thermomonosporaceae). (B) Clusters of orthologous genes shared among the six phylogenetically related species that make up the Thermomonosporaceae family. The draft genome of *Thermomonospora* sp. CIT 1 (**Bact1**) shares 597 clusters of orthologous genes with *T. curvata* DSM 43183 (**Bact2**), possessing 18 clusters that are only present in the draft genome of *Thermomonospora* sp. CIT 1. (C) Total clusters of orthologous genes verified in the six species analyzed, demonstrating that the six bacteria share a total of 132 clusters of orthologous genes among themselves. We retrieved from GenBank the nucleotide sequences of the *23S rRNA*, *recA*, *dnaK*, *dnaJ*, *dnaJ*, *atpD* and, *rpoB* genes from 19 species of *Nocardiopsis*, nine species of *Actinomadura*, and two species of *Termomonospora*. The circles found at the Maximum Likelihood phylogenetic tree nodes represent the bootstrap support value obtained for each branch. Filled black circles represent bootstrap between 90-100%; filled dark gray circles represent bootstrap between 70-89%; filled light gray circles represent bootstrap between 50-69%. **Bact1:** draft genoma CIT 1; **Bact2:** *Thermomonospora curvata* strain DSM 43183 (Thermomonosporaceae); **Bact3:** *Actinomadura rubrobrunea* strain NBRC 15275; **Bact4:** *Thermomonospora chromogena* strain DSM 43794; **Bact5:** *Actinomadura hibisca* strain NBRC 15177; **Bact6:** *Actinomadura kijaniata* strain NBRC 14229

**Table 1:** Probability pairwise comparison between the circular genome *Thermomonospora curvata* DSM 43183 and other 14 genomes of Actinobacteria.

Strain	Genome (bp)	%GC	Gene	%ANIb	%ANIm	%Tetra
^1^genome CIT 1	5,460,082	71.80	4,906	99.45 (90.79)^2^	99.62 (95.28)	99.99
*Thermomonospora chromogena* DSM 43794	5,988,017	71.00	5,122	73.15 (27.17)	83.75 (8.20)	95.57
*Actinomadura atramentaria* DSM 43919	6,709,087	73.70	6,288	75.19 (37.30)	84.27 (14.23)	87.08
*Actinomadura chibensis* NBRC 106107	9,408,892	72.80	8,425	75.64 (41.77)	84.07 (16.18)	88.19
*Actinomadura formosensis* NBRC 14204	7,079,728	71.60	6,658	76.02 (40.35)	84.29 (17.30)	92.31
*Actinomadura hibisca* NBRC 15177	9,023,974	72.40	8,470	76.78 (42.49)	84.65 (23.49)	94.30
*Actinomadura kijaniata* NBRC 14229	10,258,935	73.20	9,846	76.91 (41.05)	84.57 (22.93)	95.17
*Actinomadura latina* NBRC 106108	7,867,648	72.00	7,275	75.98 (42.07)	84.07 (18.27)	92.05
*Actinomadura macra* NBRC 14102	9,046,351	70.70	8,032	75.46 (39.83)	84.18 (15.60)	92.24
*Actinomadura rifamycini* DSM 43936	8,385,217	74.10	7,628	75.88 (41.49)	84.25 (16.94)	89.40
*Actinomadura rubrobrunea* NBRC 15275	6,724,167	72.80	5,901	77.62 (39.24)	85.12 (23.98)	95.12
*Nocardiopsis alba* ATCC BAA-2165	5,848,211	69.70	5,103	71.09 (21.50)	83.4 (4.11)	87.35
*Nocardiopsis alkaliphila* YIM 80379	5,209,552	67.50	4,722	70.78 (19.88)	83.14 (3.13)	81.97
*Nocardiopsis baichengensis* YIM 90130	6,404,110	73.60	5,580	72.22 (25.47)	83.37 (7.41)	94.18

1 Partial genome retrieved from metagenomic DNA sequencing data from filter cake stored for 40 days.

2 Percentage of total bases of the analyzed genome that aligned against the genome of T. curvata DSM 43183.

The membrane transporter cluster has many unknown functions, but one of its most prominent activities is to transport molecules of hexoses (D-mannose and D-glucose) and pentoses (L-arabinose) (Beisel and Afroz, 2016). Thus, these characteristics demonstrate that these monosaccharide molecules are derived from the deconstruction of pectin, hemicellulose, and cellulose (Vries and Visser, 2001), which may denote certain specialization of these bacteria in deconstructing (Figure 1) and capturing these molecules to use them in their metabolism.

With almost double the number of genes related to lignocellulose depolymerization in relation to the genome of *Thermomonospora curvata* DSM 43183 (Thermomonosporaceae) (Tables S3 and S4), the draft genome CIT 1 presented genes that can act on pectin and many structures of lignocellulose (Figure 1A-D). Among the specific genes of the draft genome CIT 1 that have catalytic activity on lignin, hemicellulose (galactomannan and galactoglucomannan), and starch structures are the peroxidase, arabinanase (Figures 1B and 1D), and α-amylase (Table S3). Representing peroxidases, AAs often act in synergy with the GHs during the phases of depolymerization of lignocellulosic materials, acting on lignin and producing a set of heterogeneous aromatic compounds, which are capable of being metabolized by the microorganisms (Cragg *et al.*, 2015). The α-amylases have as specific substrate, the polysaccharide starch, providing small units of glucose and maltose (Gopinath *et al.*, 2017).

The identification of many genes and metabolic pathways related to depolymerization of plant biomass, together with the identification of remaining lignocellulosic material in the filter cake, suggest that the draft genome CIT 1 has a complete set of mechanisms that can act on the deconstruction of carbohydrate polymers (Table S3 and Figure 1A-D). These mechanisms favor the release of sugars (monosaccharides, disaccharides, and oligosaccharides) that can be used as a source of energy (Vries and Visser, 2001).

Phylogenetic analysis showed that the draft genome CIT 1 clusters in the clade of *Thermomonospora curvata* DSM 43183 (Thermomonosporaceae) (Figure 2A). This result was supported by analyses of orthologous genes with Ortho Venn (Figure 2B) and ANI and Tetra nucleotide of JspeciesWS (Table 1). In addition, this taxonomic classification is in line with what was expected for this bacterial family (Kroppenstedt and Goodfellow, 2006). We observed the formation of a monophyletic pair between the draft genome CIT 1 and *T. curvata* DSM 43183, whereas there is formation of paraphyletic groups in the branches representing the species of *Actinomadura* and between *T. chromogena* and *A. rubrobrunea* (Figure 2A). This difficulty in increasing the resolution of the phylogenetic classification is related to the lack of data available in the public databases, since the species of *T. curvata* have only one complete genome and two partial genomes published in GenBank so far (November 2017, including the draft genome *Thermomonospora* sp. CIT 1 of this study).

The storage of the filter cake for long periods causes changes in its characteristics, such as loss of moisture and nutrients. In part, these modifications are due to the microbial activity in the natural substrate. The consumption of nutrients gives rise to a recalcitrant environment that increases the microbial competition and favors the occurrence of horizontal gene transfer. This mechanism may have conferred adaptive advantages for the microbial species under study from CIT 1.

The results of the predictions discussed here have not yet been extensively explored in the genomes of *Thermomonospora* species published to date. Our findings are relevant to direct research involving the production of bioethanol since the analysis of the draft genome of *Thermomonospora* sp. CIT 1 (Thermomonosporaceae) extracted from the filter cake revealed that this bacterium has almost double the number of genes that participate in the release of disaccharides and monosaccharides in relation to the genome of *T. curvata* DSM 43183. These genes can act on the main constituents of hemicellulose (galactomannan and galactoglucomannan), lignin, cellulose, starch, and pectin.

## Supplementary material

The following materials are available online for this article:

Table S1 Scaffolds unspecific for *Thermomonospora* sp. identified with blastn in the draft genome CIT 1 dataset after separation of genomic clusters with Maxbin2.

Table S2 Comparison between the partial draft genome CIT 1 statistics and strain DSM 43183 using Quast v.4.61.

Table S3 Identifications of enzymes with activity on carbohydrate structures present in the draft genome CIT 1 recovered from metagenomic sequencing of filter cake.

Table S4 Identifications of enzymes with activity on carbohydrate structures present in the circular genome of *Thermomonospora curvata* DSM 43183 (Thermomonosporaceae)
