# Supplementary material for: Draft genome of Thermomonospora sp. CIT 1 (Thermomonosporaceae) and in silico evidence of its functional role in filter cake biomass deconstruction
Source: Genet Mol Biol. 2019 Mar 11;42(1):145–50. doi: 10.1590/1678-4685-GMB-2017-0376 (PMC6428131; doi:10.1590/1678-4685-GMB-2017-0376)
Supplement: Supplementary file 1 [file 1415-4757-GMB-1678-4685-GMB-2017-0376-suppl1.pdf]

Genetics and Molecular Biology

Suplementary material to: Draft genome of *Thermomonospora* sp. CIT 1 (Thermomonosporaceae) and *in silico* evidence of its functional role in filter cake biomass deconstruction

Table S1 - Scaffolds unspecific for *Thermomonospora* sp. identified with blastn in the draft genome CIT 1 dataset after separation of genomic clusters with Maxbin2.

Scaffold	No. pb	Genus and specie	Query (%)	E.value	Identity (%)	Accession
scaffold_685	19,261	*Frankia* sp.	12	0.00E+00	75	CP000249
scaffold_818	16,010	*Thermobispora bispora*	54	0.00E+00	74	CP001874
scaffold_2995	14,612	*Actinoplanes missouriensis*	5	5.00E-72	74	AP012319
scaffold_1582	13,070	*Streptomyces albus*	0	1.80E-01	97	CP010519
scaffold_1843	7,790	*Thermomonospora curvata* DSM 43183	1	3.00E-17	85	CP001738
scaffold_2081	7,753	*Streptomyces hygroscopicus*	0	1.10E-01	100	CP003720
scaffold_2194	6,780	*Caulobacter crescentus*	0	9.40E-02	100	CP001340
scaffold_2532	6,419	*Streptomyces phage*	15	0.00E+00	80	JX182371
scaffold_3280	5,809	*Ralstonia solanacearum*	1	1.00E-04	79	CP002819
scaffold_7666	5,807	*Streptomyces* sp.	0	6.00E-03	100	CP011799
scaffold_2837	5,668	*Salinispora arenicola*	5	1.00E-20	74	CP000850
scaffold_9796	5,464	*Streptosporangium roseum*	14	2.00E-126	78	CP001814
scaffold_2909	5,450	[1]-	-	-	-	-
scaffold_2999	5,324	-	-	-	-	-
scaffold_3122	5,160	*Xanthomonas citri*	45	0.00E+00	73	CP009031
scaffold_3149	5,126	*Intrasporangium calvum*	0	3.00E-06	100	CP002343
scaffold_3200	5,080	*Streptomyces pristinaespiralis*	5	1.00E-43	80	CP011340
scaffold_3237	5,041	*Verrucosispora maris*	1	5.00E-13	93	CP002638

scaffold_3484	4,809	*Thermobispora bispora*	1	4.00E-09	91	CP001874
scaffold_8216	4,582	*Streptomyces* sp.	4	2.00E-26	78	CP011522
scaffold_3791	4,556	*Thermomonospora curvata* DSM 43183	10	4.00E-148	95	CP001738
scaffold_3954	4,435	*Streptomyces* sp.	4	4.00E-19	76	CP011799
scaffold_4214	4,259	*Streptomyces* sp.	0	5.90E-02	100	CP009754
scaffold_4308	4,201	-	-	-	-	-
scaffold_13567	4,124	PREDICTED: *Athalia rosae*	20	0.00E+00	84	XM_012405547
scaffold_5303	3,718	*Nocardia brasiliensis*	0	1.40E-02	100	CP003876
scaffold_5482	3,651	*Verrucosispora maris*	25	3.00E-88	75	CP002638
scaffold_5697	3,576	*Alloactinosynnema* sp.	3	5.00E-22	85	LN850107
scaffold_5725	3,566	*Thermomonospora curvata* DSM 43183	0	3.00E-04	100	CP001738
scaffold_8500	3,476	-	-	-	-	-
scaffold_6061	3,454	-	-	-	-	-
scaffold_9266	3,014	-	-	-	-	-
scaffold_8321	2,923	*Verrucosispora maris*	42	9.00E-123	74	CP002638
scaffold_8720	2,855	PREDICTED: *Aplysia californica*	0	4.00E-02	100	XM_013083349
scaffold_15835	2,830	-	-	-	-	-
scaffold_9954	2,665	*Actinoplanes* sp.	1	8.00E-04	100	CP005929
scaffold_15643	2,654	*Arthrobacter* sp.	1	2.00E-04	100	CP014196
scaffold_10270	2,622	-	-	-	-	-
scaffold_43456	2,551	*Comamonadaceae bacterium*	1	1.00E-02	100	AP014569
scaffold_11126	2,517	*Actinosynnema mirum*	19	2.00E-25	73	CP001630
scaffold_29869	2,500	*Micromonospora* sp.	49	0.00E+00	97	EU119229
scaffold_21038	2,482	-	-	-	-	-
scaffold_22158	2,445	-	-	-	-	-
scaffold_24206	2,336	-	-	-	-	-
scaffold_13570	2,268	-	-	-	-	-
scaffold_37494	2,257	-	-	-	-	-

scaffold_13709	2,256	-	-	-	-	-
scaffold_13807	2,249	-	-	-	-	-
scaffold_15484	2,120	-	-	-	-	-
scaffold_16670	2,039	*Streptomyces vietnamensis*	1	2.80E-02	100	CP010407
scaffold_16933	2,022	-	-	-	-	-
scaffold_18454	1,931	-	-	-	-	-
scaffold_18613	1,922	-	-	-	-	-
scaffold_18992	1,901	*Nocardia farcinica*	3	2.00E-14	90	AP006618
scaffold_19802	1,860	*Conexibacter woesei*	2	2.00E-03	91	CP001854
scaffold_20624	1,818	-	-	-	-	-
scaffold_22110	1,750	Uncultured bacterium	2	3.00E-11	100	KP438066
scaffold_44639	1,698	-	-	-	-	-
scaffold_23683	1,684	-	-	-	-	-
scaffold_33251	1,660	*Streptomyces glaucescens*	1	6.00E-03	100	CP009438
scaffold_37229	1,660	-	-	-	-	-
scaffold_25380	1,622	*Streptomyces natalensis*	58	3.00E-96	74	AY944249
scaffold_27113	1,565	-	-	-	-	-
scaffold_27740	1,545	-	-	-	-	-
scaffold_69127	1,527	-	-	-	-	-
scaffold_28337	1,526	*Excellospora viridilutea*	58	0.00E+00	94	AF134087
scaffold_43312	1,499	-	-	-	-	-
scaffold_29741	1,485	*Streptosporangium roseum*	60	1.00E-128	77	CP001814
scaffold_30083	1,476	-	-	-	-	-
scaffold_30212	1,472	*Streptomyces* sp.	1	2.00E-02	100	CP011522
scaffold_30692	1,458	-	-	-	-	-
scaffold_42965	1,445	-	-	-	-	-
scaffold_87701	1,395	-	-	-	-	-
scaffold_33646	1,385	-	-	-	-	-

scaffold_34287	1,369	-	-	-	-	-
scaffold_50931	1,362	-	-	-	-	-
scaffold_34852	1,356	*Streptomyces vietnamensis*	56	2.00E-71	74	CP010407
scaffold_77555	1,334	-	-	-	-	-
scaffold_50515	1,332	-	-	-	-	-
scaffold_36962	1,309	-	-	-	-	-
scaffold_84632	1,273	-	-	-	-	-
scaffold_39169	1,266	-	-	-	-	-
scaffold_39899	1,252	-	-	-	-	-
scaffold_40553	1,240	-	-	-	-	-
scaffold_41504	1,222	-	-	-	-	-
scaffold_43022	1,196	*Thermomonospora curvata* DSM 43183	10	2.00E-51	98	CP001738
scaffold_43579	1,187	-	-	-	-	-
scaffold_91335	1,159	-	-	-	-	-
scaffold_47904	1,121	-	-	-	-	-
scaffold_48149	1,117	-	-	-	-	-
scaffold_65964	1,100	-	-	-	-	-
scaffold_50031	1,091	-	-	-	-	-
scaffold_50378	1,086	-	-	-	-	-
scaffold_51727	1,070	-	-	-	-	-
scaffold_79301	1,066	-	-	-	-	-
scaffold_52778	1,056	-	-	-	-	-
scaffold_53819	1,044	-	-	-	-	-
scaffold_54730	1,033	-	-	-	-	-
scaffold_54845	1,032	*Streptomyces* sp.	25	2.00E-50	81	CP011522
scaffold_54960	1,030	*Kibdelosporangium phytohabitans*	97	4.00E-137	76	CP012752
scaffold_56353	1,014	*Bordetella* sp.	2	1.00E-03	100	CP013111

[1]After searching for similarity between sequences with blastn against the non-redundant nucleotide database, no results were found.
