# Supplementary material for: Draft genome of Thermomonospora sp. CIT 1 (Thermomonosporaceae) and in silico evidence of its functional role in filter cake biomass deconstruction
Source: Genet Mol Biol. 2019 Mar 11;42(1):145–50. doi: 10.1590/1678-4685-GMB-2017-0376 (PMC6428131; doi:10.1590/1678-4685-GMB-2017-0376)
Supplement: Supplementary file 2 [file 1415-4757-GMB-1678-4685-GMB-2017-0376-suppl2.pdf]

Genetics and Molecular Biology

Suplementary material to: Draft genome of *Thermomonospora* sp. CIT 1 (Thermomonosporaceae) and *in silico* evidence of its functional role in filter cake biomass deconstruction

Table S2 - Comparison between the partial draft genome CIT 1 statistics and strain DSM 43183 using Quast v.4.61. The data used for this comparison were obtained from the RAST platform and from the GenBank database.

Genome Statistics	Genome draft CIT 1	Genome reference[2]
**Without alignment genome reference (Quast)**		
Number of scaffolds	29	1
Total length scaffold (bp)	5,460,082	5,639,016
Total length scaffold (≥ 10,000 bp)	5,434,187 (23 scaffolds)	[3]-
Total length scaffold (≥ 25,000 bp)	5,418,252 (22 scaffolds)	-
Total length scaffold (≥ 50,000 bp)	5,340,441 (20 scaffolds)	-
Largest scaffold size (bp)	743,486	-
Median scaffold size (bp)	130,971	-
Shortest scaffold size (bp)	1,410	-
**With alignment genome reference (Quast)**		
Genome fraction (%)	94.68	-
Duplication ratio	1.002	-
Relocations	17	-
Misassembled scaffolds	11	-
Mismatches per 100,000 pb	243.43	-
Indels per 100,000 bp	16.61	-
N's per 100,000 bp	2.38	-
**Data obtained from RAST[4] and GenBank[5]**		
N50 size (bp)	373,446	-
L50 value	6	1
GC%	71.8	71.6
Number genes	4,906	4,941
Number proteins	4,834	4,779

[1] Detailed analysis of the supplementary material

[2] *Thermomonospora curvata* DSM 43183 (CP001738)

[3] Data not relevant for comparative analysis

[4] Only for the draft genome CIT 1

[5] Only for the draft genome of the *T. curvata* DSM 43183
