# Supplementary material for: Draft genome of Thermomonospora sp. CIT 1 (Thermomonosporaceae) and in silico evidence of its functional role in filter cake biomass deconstruction
Source: Genet Mol Biol. 2019 Mar 11;42(1):145–50. doi: 10.1590/1678-4685-GMB-2017-0376 (PMC6428131; doi:10.1590/1678-4685-GMB-2017-0376)
Supplement: Supplementary file 4 [file 1415-4757-GMB-1678-4685-GMB-2017-0376-suppl4.pdf]

Genetics and Molecular Biology

Suplementary material to: Draft genome of *Thermomonospora* sp. CIT 1 (Thermomonosporaceae) and *in silico* evidence of its functional role in filter cake biomass deconstruction

Table S4 - Identifications of enzymes with activity on carbohydrate structures present in the circular genome of *Thermomonospora curvata* DSM 43183 (Thermomonosporaceae). Predictions were performed with dbCAN online, following for blastp confirmation against the non-redundant NCBI protein database of the occurrence of predicted protein-like sequence deposition.

ORF LENGTH	Search results for dbCAN conserved domains		BLASTP RESULTS				
	CAZy FAMILY	ID CAZy	ENZYME NAME (NCBI)	QUERY COVER	E-VALUE	IDENTIT Y	ACCESSION
517	AA3.hmm	glucose-methanol-choline (GMC)	mycofactocin system GMC family oxidoreductase MftG	100	0.00E+00	100	WP_012852714
528	AA4.hmm	vanillyl-alcohol oxidase (EC 1.1.3.38)	FAD-binding oxidoreductase	99	0.00E+00	76	WP_067912508
209	AA6.hmm	1,4-benzoquinone reductase (EC. 1.6.5.6)	NAD(P)H dehydrogenase (quinone)	99	4.00E-128	88	SEG76937
186	AA6.hmm	1,4-benzoquinone reductase (EC. 1.6.5.6)	NAD(P)H-dependent FMN reductase	100	2.00E-84	70	SEG82383
411	AA7.hmm	glycolate oxidase (EC 1.1.3.15)	glycolate oxidase FAD binding subunit	98	0.00E+00	77	SEG60993
482	AA7.hmm	glycolate oxidase (EC 1.1.3.15)	glycolate oxidase	100	0.00E+00	88	SEG60983
459	AA7.hmm	[1]-	FAD-binding oxidoreductase	99	0.00E+00	72	WP_091378734
212	AA10.hmm	lytic polysaccharide monooxygenases (LPMOs)	chitin-binding protein	100	7.00E-86	73	WP_079318887
505	CBM35.hmm	CBM35	CBM35+GH43	96	3.00E-172	57	SEG79516
943	CBM44.hmm	-	PKD domain containing protein	95	0.00E+00	66	WP_083597587
736	CBM48.hmm	-	1,4-alpha-glucan branching enzyme	98	0.00E+00	86	SEF80265
92	CBM48.hmm	-	hypothetical protein	98	7.00E-45	75	WP_030510813
292	CE1.hmm	cinnamoyl esterase (EC 3.1.1.-)	lipase	100	0.00E+00	100	WP_012850775

ORF LENGTH	Search results for dbCAN conserved domains		BLASTP RESULTS				
	CAZy FAMILY	ID CAZy	ENZYME NAME (NCBI)	QUERY COVER	E-VALUE	IDENTIT Y	ACCESSION
365	CE1.hmm	-	hypothetical protein	99	0.00E+00	99	WP_052305466
341	CE1.hmm	S-formylglutathione hydrolase (EC 3.1.2.12)	S-formylglutathione hydrolase FrmB	99	0.00E+00	78	SEG71481
284	CE1.hmm	esterase	alpha/beta hydrolase	100	0.00E+00	99	WP_041439411
336	CE1.hmm	-	polyhydroxybutyrate depolymerase	96	2.00E-171	76	SEF89583
317	CE1.hmm	esterase	Pimeloyl-ACP methyl ester carboxylesterase	100	0.00E+00	85	SEG14212
285	CE4.hmm	-	polysaccharide deacetylase family protein	100	0.00E+00	100	WP_012855020
267	CE4.hmm	-	polysaccharide deacetylase	100	0.00E+00	100	WP_012851516
264	CE4.hmm	-	polysaccharide deacetylase	100	0.00E+00	100	WP_012853908
285	CE4.hmm	-	polysaccharide deacetylase family protein	100	0.00E+00	100	WP_012853293
241	CE5.hmm	cutinase (EC 3.1.1.74)	cutinase	100	9.00E-176	100	ACY97091
234	CE10.hmm	esterase	alpha/beta hydrolase fold-3 domain-containing protein	100	2.00E-158	100	WP_012853073
349	CE10.hmm	-	hypothetical protein	98	0.00E+00	99	WP_083789966
497	CE10.hmm	-	peptidase S15	100	0.00E+00	100	WP_012852916
668	CE10.hmm	-	S9 family peptidase	100	0.00E+00	100	WP_012853613
343	CE10.hmm	esterase	alpha/beta hydrolase	100	0.00E+00	100	WP_012854310
317	CE10.hmm	esterase	alpha/beta hydrolase	100	0.00E+00	99	WP_083789919
335	CE10.hmm	esterase	alpha/beta hydrolase	100	0.00E+00	100	WP_012851463
303	CE14.hmm	mycothiol S-conjugate amidase (EC 3.5.1.-)	mycothiol conjugate amidase Mca	97	0.00E+00	100	WP_041439331
271	CE14.hmm	N-acetyl-1-D-myo-inosityl-2-amino-2-deoxy-α-D-glucopyranoside deacetylase (EC 3.5.1.89)	GlcNAc-PI de-N-acetylase	100	0.00E+00	100	WP_041441415
297	CE14.hmm	N-acetyl-1-D-myo-inosityl-2-amino-2-deoxy-α-D-glucopyranoside deacetylase (EC 3.5.1.89)	1D-myo-inositol 2-acetamido-2-deoxy-alpha-D-glucopyranoside deacetylase	100	0.00E+00	100	WP_012851500
411	GH1.hmm	β-glucosidase (EC 3.2.1.21)	β-glucosidase	100	0.00E+00	100	WP_012852534

ORF LENGTH	Search results for dbCAN conserved domains		BLASTP RESULTS				
	CAZy FAMILY	ID CAZy	ENZYME NAME (NCBI)	QUERY COVER	E-VALUE	IDENTITY	ACCESSION
447	GH1.hmm	β-glucosidase (EC 3.2.1.21)	β-glucosidase	100	0.00E+00	100	WP_012855073
427	GH4.hmm	-	glycoside hydrolase family 4	100	0.00E+00	100	WP_012854171
618	GH5_43.hmm	glucosylceramidase (EC 3.2.1.45)	endoglycosylceramidase	100	0.00E+00	100	WP_012851730
419	GH6.hmm	-	glycoside hydrolase family 6	100	0.00E+00	100	WP_012852096
601	GH13_16.hmm	-	maltose α-D-glucosyltransferase	100	0.00E+00	99	WP_083790013
684	GH13_3.hmm	α-1,4-glucan: phosphate α-maltosyltransferase (EC 2.4.99.16)	α-1,4-glucan--maltose-1-phosphate maltosyltransferase	100	0.00E+00	100	WP_012853902
736	GH13_9.hmm	-	1,4-α-glucan-branching protein	100	0.00E+00	100	WP_012853921
629	GH15.hmm	-	glycoside hydrolase family 15	100	0.00E+00	100	WP_012853541
484	GH15.hmm	-	glycoside hydrolase family 15	100	0.00E+00	100	WP_012854532
378	GH23.hmm	peptidoglycan lyase (EC 4.2.2.n1) also known in the literature as peptidoglycan lytic transglycosylase; chitinase (EC 3.2.1.14)	lytic transglycosylase catalytic subunit	100	0.00E+00	100	WP_012852049
349	GH23.hmm	-	NLP/P60 protein	100	0.00E+00	100	WP_012851472
266	GH23.hmm	peptidoglycan lyase (EC 4.2.2.n1) also known in the literature as peptidoglycan lytic transglycosylase; chitinase (EC 3.2.1.14)	transglycosylase domain-containing protein	100	0.00E+00	100	WP_012851352
272	GH23.hmm	-	hypothetical protein	100	0.00E+00	100	WP_012851447
380	GH25.hmm	-	glycoside hydrolase family 25	100	0.00E+00	100	WP_012850936
297	GH25.hmm	-	glycoside hydrolase family 25	100	0.00E+00	100	WP_012852705
335	GH26.hmm	-	hypothetical protein	100	0.00E+00	100	WP_012851356
334	GH26.hmm	β-mannanase (EC 3.2.1.78)	beta-mannanase-like protein	100	0.00E+00	100	WP_012854497
465	GH30.hmm	-	hypothetical protein	100	0.00E+00	100	WP_012853919
505	GH43_30.hmm	-	glycoside hydrolase family 43	100	0.00E+00	100	WP_012851007
456	GH63.hmm	-	hypothetical protein	100	0.00E+00	100	WP_012852353
807	GH65.hmm	-	glycoside hydrolase family 65	100	0.00E+00	100	WP_012855093

ORF LENGTH	Search results for dbCAN conserved domains		BLASTP RESULTS				
	CAZy FAMILY	ID CAZy	ENZYME NAME (NCBI)	QUERY COVER	E-VALUE	IDENTITY	ACCESSION
810	GH65.hmm	-	glycosyl hydrolase	100	0.00E+00	100	WP_012853146
794	GH65.hmm	-	glycoside hydrolase family 65	100	0.00E+00	100	WP_012853851
717	GH77.hmm	amylomaltase or 4-α-glucanotransferase (EC 2.4.1.25)	4-alpha-glucanotransferase	100	0.00E+00	100	WP_012851713
283	GH117.hmm	-	hypothetical protein	100	0.00E+00	100	WP_012851745
380	GT1.hmm	-	DUF1205 domain-containing protein	98	0.00E+00	100	WP_083789857
418	GT1.hmm	-	glycosyltransferase	100	0.00E+00	100	WP_012855060
272	GT2.hmm	-	glycosyltransferase family 2 protein	100	0.00E+00	100	WP_012853905
337	GT2.hmm	-	glycosyltransferase family 2 protein	100	0.00E+00	100	WP_012853907
157	GT2.hmm	-	CDP-glycerol:glycerophosphate glycerophosphotransferase	100	0.00E+00	100	WP_012851021
248	GT2.hmm	-	polyprenol monophosphomannose synthase	100	1.00E-178	100	WP_012852699
807	GT2.hmm	-	Acyl-CoA synthetase (AMP-forming)/AMP-acid ligase II	97	0.00E+00	86	SEG89133
301	GT2.hmm	-	glycosyltransferase family 2 protein	100	0.00E+00	100	WP_012851375
537	GT2.hmm	-	glycosyl transferase family 2	87	0.00E+00	99	WP_041442530
306	GT2.hmm	-	glycosyl transferase	95	0.00E+00	90	SEG79500
321	GT2.hmm	-	glycosyltransferase family 2 protein	100	0.00E+00	100	WP_012851019
315	GT2.hmm	-	glycosyl transferase	100	0.00E+00	100	WP_012851009
1148	GT2.hmm	-	CDP-glycerol--poly(glycerophosphate) glycerophosphotransferase	100	0.00E+00	100	WP_012854410
658	GT2.hmm	-	glycosyl transferase	100	0.00E+00	100	WP_012851015
430	GT2.hmm	-	glycosyltransferase family 2 protein	98	0.00E+00	100	WP_083789964
381	GT4.hmm	-	glycosyl transferase	100	0.00E+00	100	WP_012853078

ORF LENGTH	Search results for dbCAN conserved domains		BLASTP RESULTS				
	CAZy FAMILY	ID CAZy	ENZYME NAME (NCBI)	QUERY COVER	E-VALUE	IDENTITY	ACCESSION
388	GT4.hmm	-	glycosyltransferase family 1 protein	100	0.00E+00	100	WP_012852452
381	GT4.hmm	-	glycosyltransferase family 1 protein	98	0.00E+00	99	WP_041441526
443	GT4.hmm	-	glycosyltransferase family 4 protein	100	0.00E+00	100	WP_012852806
431	GT4.hmm	-	D-inositol-3-phosphate glycosyltransferase	100	0.00E+00	100	WP_012851072
452	GT4.hmm	-	glycosyltransferase family 1 protein	100	0.00E+00	100	WP_012853975
404	GT4.hmm	-	glycosyltransferase family 1 protein	100	0.00E+00	100	WP_012850472
290	GT14.hmm	-	glycosyl transferase family 14	100	0.00E+00	100	WP_012851388
470	GT20.hmm	α,α-trehalose-phosphate synthase [UDP-forming] (EC 2.4.1.15)	trehalose-6-phosphate synthase	100	0.00E+00	99	WP_083789822
595	GT39.hmm	-	hypothetical protein	100	0.00E+00	100	WP_012851347
773	GT51.hmm	-	glycosyl transferase family 51	100	0.00E+00	100	WP_012851396
265	GT81.hmm	-	glycosyl transferase family 2	100	0.00E+00	100	WP_012850508
504	GT83.hmm	-	hypothetical protein	100	0.00E+00	100	WP_012854498
429	GT87.hmm	-	DUF2029 domain-containing protein	100	0.00E+00	100	WP_012850510
404	GT94.hmm	-	glycosyltransferase family 1 protein	100	0.00E+00	100	WP_012850472
327	PL1.hmm	pectate lyase (EC 4.2.2.2)	pectate lyase	100	0.00E+00	100	WP_012852783
616	PL22_1.hmm	-	hypothetical protein	100	0.00E+00	99	WP_086014605

[1] The name of the enzyme differs between the CAZy databases and non-redundant NCBI proteins.
